# The mediating roles of gut microbiota in the associations of air pollution and meteorological factors with metabolic syndrome

**DOI:** 10.1002/imo2.70099

**Published:** 2026-05-07

**Authors:** Yixiang Huang, Wei Wu, Yuan Zheng, Hongwei Tu, Yiping Duan, Qijiong Zhu, Zhiqing Chen, Siwen Yu, Yayi Li, Wan Peng, Wenjun Ma, Tao Liu

**Affiliations:** ^1^ Department of Public Health and Preventive Medicine, School of Medicine Jinan University Guangzhou China; ^2^ China Greater Bay Area Research Center of Environmental Health, School of Medicine Jinan University Guangzhou China; ^3^ Key Laboratory of Viral Pathogenesis & Infection Prevention and Control Jinan University, Ministry of Education Guangzhou China; ^4^ Guangdong Provincial Institute of Public Health Guangdong Provincial Center for Disease Control and Prevention Guangzhou China

## Abstract

We characterized distinct gut microbial community clusters associated with metabolic syndrome (MetS) and identified key genera underlying these patterns. Health‐associated clusters were dominated by beneficial genera such as *Bacteroides* and *Prevotella* with known metabolic protective functions. Environmental exposures (air pollution and temperature) were associated with gut microbiota composition, and may increase the risk of MetS. Our findings further suggest that specific bacterial genera, particularly *Megamonas* and *Megasphaera*, may statistically mediate the associations of long‐term exposure to nitrogen dioxide (NO_2_), fine particulate matter (PM_2.5_), and ambient temperature with MetS risk. Integrating environmental exposure assessment with gut microbiota profiling offers a promising strategy for metabolic syndrome prevention and precision intervention.

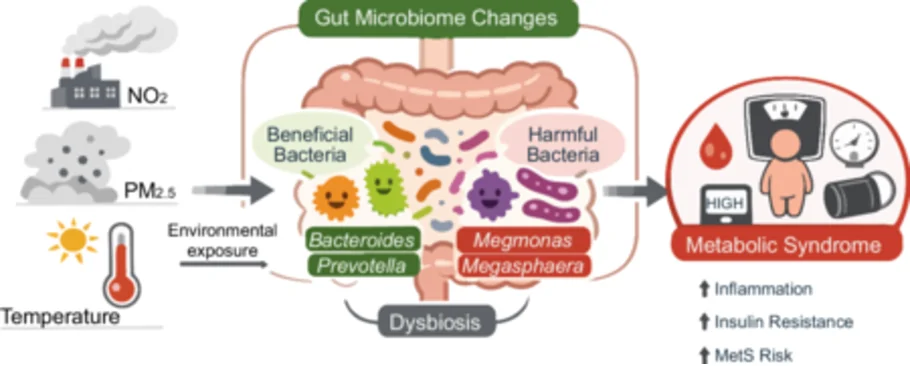

To the Editor,

Metabolic syndrome (MetS) has become a major public health concern, affecting nearly one quarter of the global population, and its global prevalence is projected to continue rising, reaching approximately 53% by 2035 [1, 2]. MetS confers increased risks of cardiovascular disease, type 2 diabetes, sleep apnea, malignancies, and all‐cause mortality [3]. In addition to genetic and lifestyle factors, accumulating evidence suggests that environmental exposures play an important role in the development of MetS [4, 5]. Long‐term exposure to air pollutants such as fine particulate matter (PM_2.5_) and nitrogen dioxide (NO_2_), as well as meteorological factors, particularly ambient temperature, has been associated with an increased risk of MetS [4, 5]. However, the biological mechanisms linking environmental exposures to MetS remain incompletely understood.

Recent studies have increasingly highlighted the gut microbiota as a key regulator of host metabolic homeostasis [6]. Gut microbial dysbiosis can impair intestinal barrier function, promote chronic low‐grade inflammation, and disturb glucose and lipid metabolism, thereby contributing to MetS [7]. Emerging evidence from the One Health Microbiome perspective further suggests that environmental stressors, including air pollution and weather fluctuations, can reshape gut microbial composition and function [8]. Nevertheless, it remains unclear whether environmentally induced alterations in the gut microbiota contribute to the development of MetS. We hypothesized that alterations in the gut microbiota may represent an important biological pathway through which environmental exposures contribute to the risk of MetS.

Using data from the Guangdong Gut Microbiome Project, we investigated the associations of long‐term exposure to NO_2_, PM_2.5_, and ambient temperature with MetS, and performed exploratory mediation analyses to assess potential indirect associations through the gut microbiota. Our study aims to provide population‐based evidence that the gut microbiota represents a key biological pathway linking environmental exposures to metabolic dysfunction.

## 1 PARTICIPANT CHARACTERISTICS AND OVERALL MICROBIOME FEATURES

Among the overall 6785 participants, 1258 individuals (18.5%) were diagnosed with MetS, and 3035 (44.7%) were males. High blood pressure (51.1%) and low high‐density lipoprotein cholesterol (HDL‐C) (26.7%) were the most prevalent MetS components, whereas elevated fasting blood glucose (15.2%) was the least frequent. Males showed slightly higher prevalence of high blood pressure and high triglycerides, whereas females had a higher prevalence of abdominal obesity (Figure S1 and Table S1). Compared with controls, MetS cases were older, had lower incomes and educational attainment, engaged in less physical activity, and had higher prevalences of smoking and alcohol consumption (Table S1). In addition, MetS cases were exposed to higher levels of NO_2_ and PM_2.5_, as well as to lower ambient temperatures (Figure S2). Based on 16S rRNA gene sequencing data, we compared the overall gut microbial composition between MetS cases and controls. At both the phylum and genus levels, the overall taxonomic profiles were broadly similar between two groups (Figure S3). To capture more subtle MetS‐related microbiome differences, we conducted downstream analyses to characterize microbiome features associated with MetS.

## 2 ASSOCIATIONS OF GUT MICROBIAL DIVERSITY, COMMUNITY CLUSTERS, AND KEY GENERA WITH METS

Compared with non‐MetS participants, those with MetS exhibited lower gut microbial alpha diversity across all indices, including Shannon, phylogenetic diversity (PD whole tree), Chao1, and observed species (all *p‐*values < 0.05; Figure 1A). An elbow plot of the within‐cluster sum of squares supported a four‐cluster solution as best capturing the underlying community structure (Figure S4). In the resulting Uniform Manifold Approximation and Projection (UMAP) ordination, the four clusters were clearly separated. Pseudotime analysis provided a data‐driven ordering of samples along the main microbiome manifold. Along this ordering, clusters differed in MetS prevalence, suggesting that variation in gut microbial community composition was aligned with differences in metabolic status across clusters (Figure 1B). To identify genera associated with cluster differentiation, we compared genus‐level abundances in each cluster. Cluster 1 was mainly enriched in *Bacteroides* and *Parabacteroides*, whereas cluster 2 was characterized by enrichment of *Prevotella* and *Desulfovibrio*. Cluster 3 showed a relatively diffuse enrichment pattern across multiple environmental or opportunistic genera, without a single strongly dominant genus, as all differences in centered log‐ratio (ΔCLR) values were < 1.5. In contrast, cluster 4 was dominated by members of the family Enterobacteriaceae, with marked overrepresentation of opportunistic taxa such as *Serratia*, *Enterobacter*, *Citrobacter*, and *Klebsiella* (Figure 1C).

**Figure 1:**
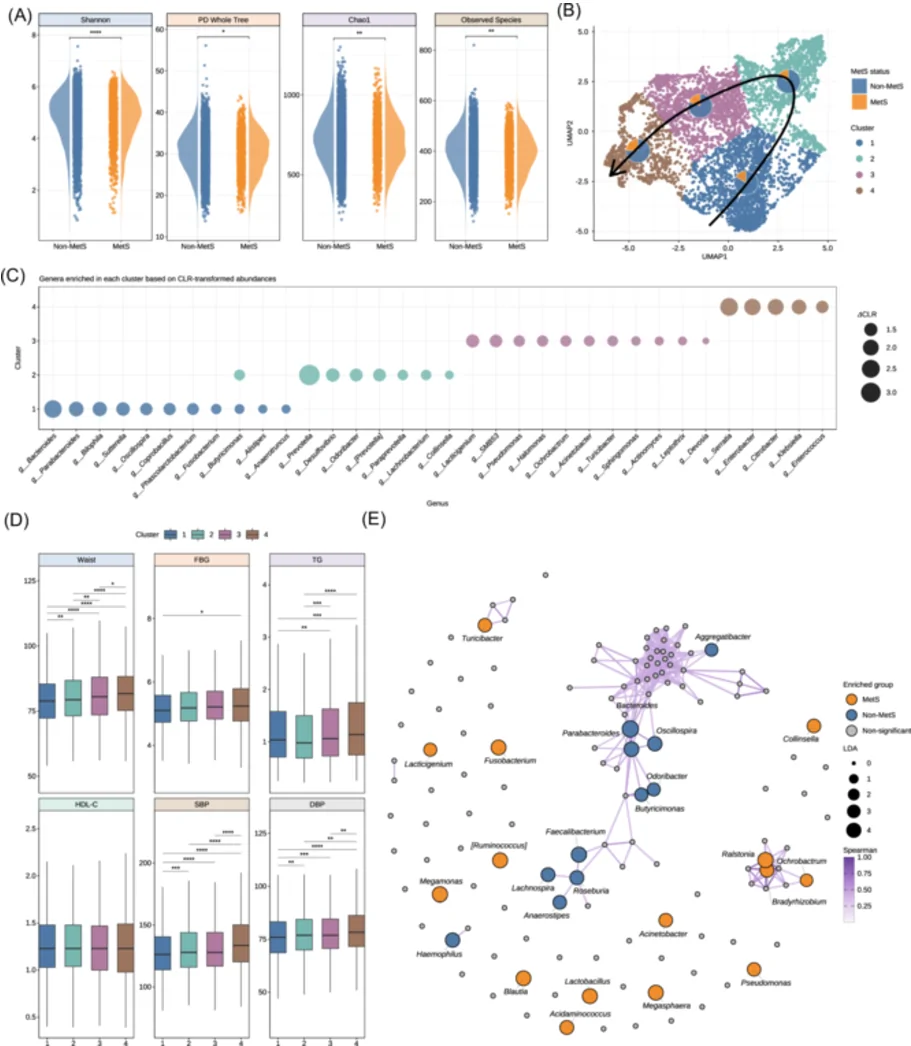
Gut microbial diversity, community structure, key genera, and clinical phenotypes in relation to MetS. (A) Differences in gut microbial α‐diversity between MetS and non‐MetS participants, with group differences assessed using the Wilcoxon rank‐sum test. (B) UMAP plot showing four data‐driven gut microbial community clusters and their distribution along the pseudotime ordering, with corresponding MetS case proportions. The black arrow indicates the inferred direction of pseudotime progression across the main microbiome manifold. (C) Bubble plot showing cluster‐enriched genera (FDR < 0.05) identified by comparing each cluster with all others, with point size reflecting ΔCLR. (D) Boxplots showing variation in MetS‐related clinical indicators across four gut microbiota‐defined clusters. (E) Network graph showing genus‐level co‐variation patterns among LEfSe‐identified genera. Edges represent positive Spearman correlations, with darker purple edges indicating stronger correlations. Correlations with Spearman's |*ρ*| < 0.4 were filtered out, and only positive correlations were retained in the final network. *p < 0.05, **p < 0.01, ***p < 0.001, ****p < 0.0001. DBP, diastolic blood pressure; FDR, false discovery rate; FBG, fasting blood glucose; HDL‐C, high‐density lipoprotein cholesterol; LEfSe, linear discriminant analysis effect size; MetS, metabolic syndrome; ΔCLR, differences in CLR‐transformed abundances; TG, triglycerides; SBP, systolic blood pressure; UMAP, Uniform Manifold Approximation and Projection; Waist, waist circumference.

We further compared MetS‐related clinical indicators across the four clusters. Participants in clusters 3 and 4 generally exhibited a more adverse clinical profile than those in clusters 1 and 2, with higher waist circumference, fasting glucose, triglycerides, and systolic and diastolic blood pressure, together with lower HDL‐C (Figure 1D). In contrast, participants in clusters 1 and 2 showed the most favorable metabolic profiles, consistent with their enrichment in *Bacteroides* and *Prevotella*. Previous studies have shown that *Bacteroides* plays an important role in maintaining glucose homeostasis and overall metabolic balance [9]. Likewise, *Prevotella*‐dominated communities are characterized by an enhanced capacity to metabolize plant‐derived carbohydrates and by robust production of short‐chain fatty acids (SCFAs) from diets rich in complex carbohydrates and dietary fibers [10]. SCFAs have been implicated in the regulation of glucose and lipid metabolism, insulin sensitivity, and inflammatory responses, which may be relevant to metabolic health [11]. We then constructed a genus‐level co‐variation network based on genera identified by linear discriminant analysis effect size (LEfSe). A dense module was mainly observed among genera enriched in the non‐MetS group, including *Bacteroides*, *Faecalibacterium*, and *Parabacteroides*, which showed predominantly positive correlations with one another. In contrast, genera enriched in the MetS group were more dispersed across the network and did not form a comparably dense module (Figure 1E).

## 3 MEDIATION OF GUT MICROBIOTA ON THE ASSOCIATIONS OF ENVIRONMENTAL EXPOSURE WITH METS

We first used univariate permutational multivariate analysis of variance (PERMANOVA) to quantify the variance in gut microbial community structure explained by host, environmental, and lifestyle factors. Geographical location explained the largest proportion of between‐sample variance in gut microbiota composition. Among non‐geographical variables, NO_2_, ambient temperature, and PM_2.5_ explained relatively large proportions of variance (ranked first, sixth, and seventh, respectively), indicating that air pollution and meteorological conditions were important correlates of gut microbiota composition (Figure 2A). These findings indicate that, beyond regional background, environmental exposures are important modifiable factors associated with gut microbial community structure.

**Figure 2:**
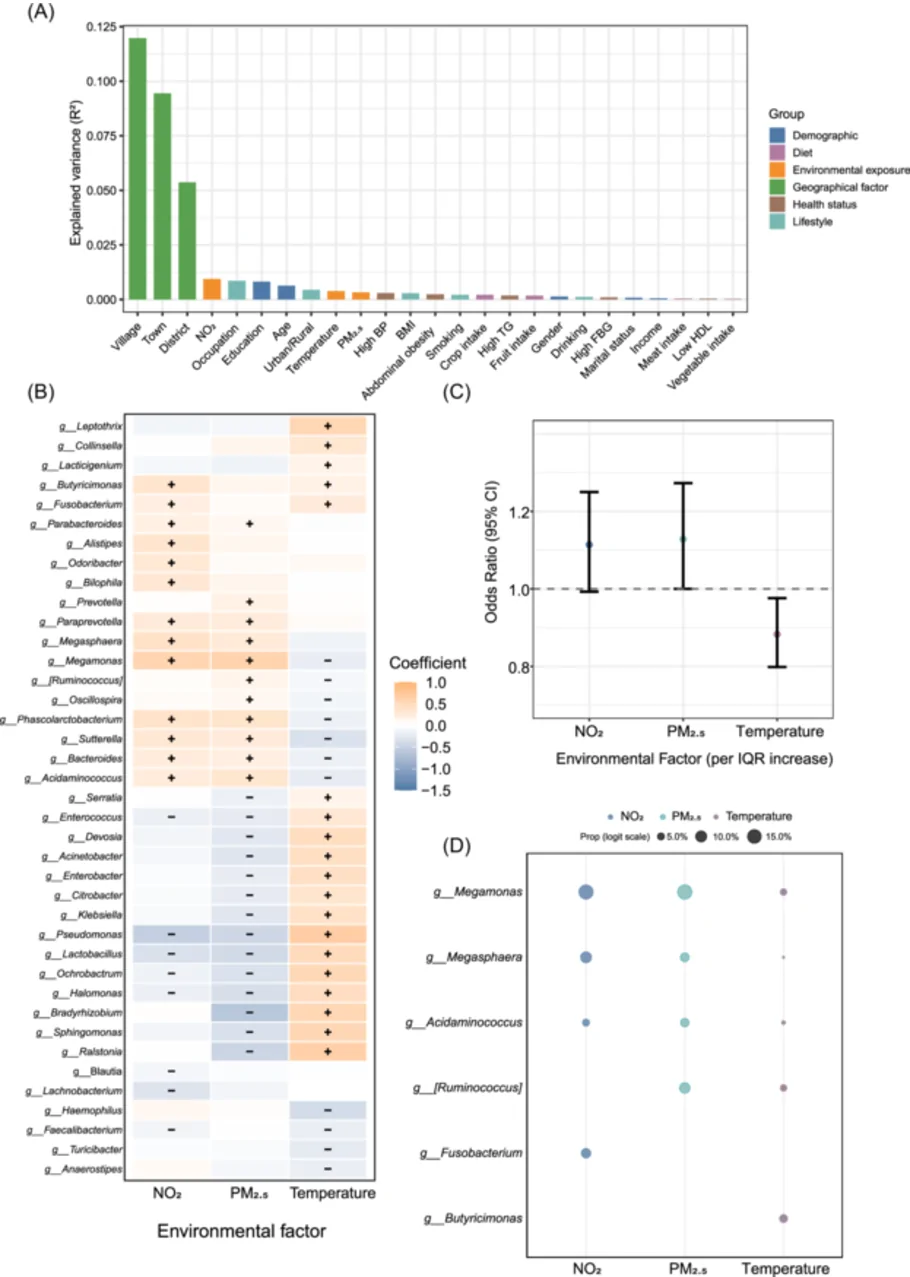
Environmental drivers of gut microbiota and their associations with metabolic syndrome through mediation by key gut microbial genera. (A) Bar plot showing PERMANOVA‐based estimates of variance in gut microbial community structure explained by geographical, environmental, demographic, lifestyle, and MetS‐related clinical factors. (B) Heat map showing associations of gut microbial genera with NO_2_, PM_2.5_, and ambient temperature. In the heat map, “+” denotes a significant positive association and “−” denotes a significant inverse association after FDR correction. (C) Associations of NO_2_, PM_2.5_, and ambient temperature with MetS. Models were adjusted for sex, age, education, race, household income, marital status, occupation, type of cooking fuel, smoking status, alcohol consumption, physical activity, urban–rural location, season of survey, and intakes of major food groups. (D) Bubble plot showing exploratory mediation signals for the associations of environmental exposures with MetS across selected gut microbial genera. Bubble size reflects the estimated mediated proportion derived from single‐mediator logistic mediation models. Because microbial genera are correlated, these scale‐dependent estimates are non‐additive and should be interpreted as exploratory screening results rather than independent contributions across genera. BMI, body mass index; BP, blood pressure; CI, confidence interval; FDR, false discovery rate; FBG, fasting blood glucose; HDL‐C, high‐density lipoprotein cholesterol; IQR, interquartile range; NO_2_, nitrogen dioxide; PERMANOVA, permutational multivariate analysis of variance; PM_2.5_, fine particulate matter; *R*², coefficient of determination; TG, triglycerides.

We next assessed associations between key microbial genera and environmental exposures, focusing on genera associated with the previously defined clusters and those identified by LEfSe. Their associations with NO_2_, PM_2.5_, and ambient temperature showed two contrasting patterns. Genera such as *Megamonas, Megasphaera, Acidaminococcus*, and *Bacteroides* were positively associated with NO_2_ and PM_2.5_ but inversely associated with ambient temperature. In contrast, genera including *Pseudomonas, Lactobacillus*, and *Ochrobactrum* generally showed negative associations with air pollutants and positive associations with ambient temperature (Figure 2B).

Given these exposure‐microbiome patterns, we further quantified the associations between environmental exposures and MetS. In logistic regression models, higher NO_2_ and PM_2.5_ concentrations were associated with increased odds of MetS, whereas higher ambient temperature was associated with lower odds of MetS: per interquartile‐range increase, the odds ratios (ORs) for MetS were 1.114 (95% confidence interval (CI): 0.993–1.250) for NO_2_, 1.128 (95% CI: 1.000–1.273) for PM_2.5_, and 0.833 (95% CI: 0.798–0.976) for ambient temperature. Quartile‐based analyses showed broadly consistent patterns, with higher quartiles of NO_2_ and PM_2.5_ generally associated with higher adjusted odds of MetS, whereas higher ambient temperature quartiles tended to be associated with lower odds (Figure 2C and Table S3). These findings are consistent with previous epidemiological evidence [12, 13, 14].

Finally, we performed exploratory statistical mediation analyses to clarify the role of the gut microbiota in the associations between environmental exposures and MetS. When gut microbial genera were specified as potential mediators, six genera (*Megamonas, Megasphaera, Ruminococcus, Acidaminococcus, Butyricimonas*, and *Fusobacterium*) showed evidence of exploratory statistical mediation for the associations of NO_2_, PM_2.5_, and ambient temperature with MetS, with a false discovery rate < 0.10 (Figure 2D and Table S4). Because genus‐level microbial features are correlated, findings from single‐mediator models should be interpreted as exploratory screening results. In addition, the estimated mediated proportions derived from the logistic mediation models are non‐additive and should be interpreted as scale‐dependent relative indirect contributions, rather than independent contributions across genera or proportions on an absolute risk scale. Among these genera, *Megamonas* showed the largest estimated mediated proportions in the logistic mediation models, with values of 15.1%, 16.1%, and 4.7% for the associations of PM_2.5_, NO_2_, and ambient temperature with MetS, respectively. In addition, *Megasphaera* showed corresponding estimated mediated proportions of 6.3%, 9.9%, and 3.1% for the associations of PM_2.5_, NO_2_, and ambient temperature with MetS, respectively. *Ruminococcus* showed estimated mediated proportions of 8.4% for the association of PM_2.5_ with MetS and 4.7% for the association of ambient temperature with MetS, whereas no evidence of mediation was observed for NO_2_. Similarly, *Acidaminococcus* showed estimated mediated proportions of 5.9% for the association between PM_2.5_ and MetS, 5.0% for the association between NO_2_ and MetS, and 3.3% for the association between ambient temperature and MetS.

These results suggest exploratory statistical mediation, indicating that gut microbiota features may account for part of the observed associations between environmental exposures and MetS, with patterns that may vary by exposure type. For air pollution, mediation was dominated by *Megamonas*, with additional contributions from *Megasphaera* and *Acidaminococcus*. Notably, *Megamonas* has been reported to harbor genes involved in myo‐inositol degradation, and experimental evidence shows that colonization with *Megamonas rupellensis* enhances intestinal lipid absorption and promotes obesity, which supports biological plausibility for a potential microbiome‐related pathway [15]. A Japanese study reported a positive association between *Megasphaera* abundance and type 2 diabetes, supporting its adverse metabolic relevance [16]. In addition, a meta‐analysis reported a higher relative abundance of *Acidaminococcus* in obese than in non‐obese adults, suggesting a potential link with adverse metabolic profiles [17]. However, direct mechanistic evidence remains limited, and its role in pollution‐related metabolic dysfunction warrants further investigation. For ambient temperature, mediation was mainly driven by *Butyricimonas, Megamonas, Ruminococcus*, and *Acidaminococcus*. Notably, *Megamonas* and *Acidaminococcus* showed consistent mediating effects for both air pollution and ambient temperature, suggesting a shared set of genera implicated in the exploratory mediation results. Prior studies have reported that *Butyricimonas* was identified as a differential genus in mice exposed to high temperature and humidity, which was further associated with altered plasma insulin and glucose levels [18]. In addition, *Ruminococcus* has been linked to altered carbohydrate fermentation, impaired gut barrier function, and chronic low‐grade inflammation, processes closely related to host energy balance and potentially sensitive to temperature‐related physiological perturbations [19]. Together, these findings suggest that specific gut microbial genera may mediate part of the metabolic impact of environmental exposures and highlight the gut microbiota as a potential target for MetS prevention and intervention.

In summary, this study found that MetS was associated with lower gut microbial α‐diversity, distinct community clusters, and differences in key genera, including enrichment of several potentially pathogenic genera. Higher long‐term exposure to NO_2_ and PM_2.5_ and lower ambient temperature were related to increased odds of MetS, and several genera, such as *Megamonas*, *Megasphaera*, *Acidaminococcus*, and *Ruminococcus*, were identified in exploratory mediation analyses as accounting for part of the associations. These findings indicate that the gut microbiota may act as a biological interface through which environmental exposures influence MetS risk.

The cross‐sectional design precludes causal inference among environmental exposures, gut microbiota, and MetS, and longitudinal and experimental studies are needed to clarify temporal and causal relationships. Residual confounding remains possible, particularly from unmeasured factors such as medication use and host genetics, which were unavailable in this study and may affect both microbiota composition and MetS. In addition, the lack of detailed information on time‐activity patterns, indoor air pollution, environmental noise, and green space may lead to exposure misclassification. Although we examined several major environmental factors, including NO_2_, PM_2.5_, and temperature, other relevant exposures were not comprehensively assessed, and the use of a 2‐year averaging window may obscure potentially relevant exposure periods. Future studies are warranted to incorporate a broader range of exposures, alongside finer‐resolution exposure assessment and alternative exposure windows. Nevertheless, integrating environmental exposures with gut microbiota profiles remains a promising strategy for elucidating MetS and may facilitate more precise risk stratification and prevention.

## AUTHOR CONTRIBUTIONS

**Yixiang Huang**: Writing—original draft; visualization; software; methodology. **Wei Wu**: Writing—review & editing; resources; data curation. **Yuan Zheng**: Writing—review & editing; data curation. **Hongwei Tu**: Writing—review & editing; methodology; resources. **Yiping Duan**: Writing—review & editing; data curation. **Qijiong Zhu**: Writing—review & editing; methodology; resources. **Zhiqing Chen**: Writing—review & editing; methodology. **Siwen Yu**: Writing—review & editing; conceptualization; project administration. **Yayi Li**: Writing—review & editing; data curation. **Wan Peng**: Writing—review & editing; software. **Wenjun Ma**: Writing—review & editing; writing—original draft; project administration; methodology; conceptualization. **Tao Liu**: Writing—review & editing; writing—original draft; project administration; methodology; conceptualization. All authors have read the final manuscript and approved it for publication.

## CONFLICT OF INTEREST STATEMENT

The authors declare no conflicts of interest.

## ETHICS STATEMENT

This study was approved by the Ethical Review Committee of the Chinese Center for Disease Control and Prevention (approval number: 201519‐B). All participants provided written informed consent.

## AI STATEMENT

The initial draft of this manuscript was polished for grammar and sentence structure using ChatGPT and Grammarly (https://app.grammarly.com/). All AI‐generated contents were meticulously reviewed and edited by the authors to ensure factual accuracy and prevent any potential AI‐induced bias. We hereby confirm that no AI tools were employed in the processes of data generation, analysis, or visual content creation.

## Supporting information


**Figure S1:** Radar plot showing the prevalence of MetS and its components in the overall population and stratified by sex.
**Figure S2:** Boxplots comparing long‐term exposure levels to PM_2.5_, NO_2_, and ambient temperature between MetS and non‐MetS participants.
**Figure S3:** Phylum and genus ‐level relative abundance of gut microbiota in non‐MetS and MetS groups, based on 16S rRNA gene sequencing.
**Figure S4:** Elbow plot for selecting the optimal number of gut microbial community clusters.
**Figure S5:** The process of long‐term exposure assessment to air pollutants.
**Figure S6:** Random forest variable‐importance ranking of six ambient air pollutants (PM_2.5_, NO_2_, CO, PM_10_, SO_2_, and O_3_) for predicting MetS.
**Figure S7:** SHAP‐based feature importance of six ambient air pollutants in the XGBoost model for predicting MetS.

**Table S1:** Prevalence of metabolic syndrome and its diagnostic components.
**Table S2:** General characteristics of study participants by MetS status.
**Table S3:** ORs and 95% CIs of the associations between environmental factors and MetS.
**Table S4:** Mediation of the associations between environmental exposures and MetS by selected gut microbial genera.
**Table S5:** Variables used in the spatiotemporal land use regression (LUR) model.
**Table S6:** The definitions of each category of included variables.

## Data Availability

The raw data for 16S rRNA gene sequences are available from the European Nucleotide Archive (ENA) under the accession code PRJEB18535 (https://www.ebi.ac.uk/ena/browser/view/PRJEB18535). All analysis data and scripts are publicly available on the GitHub website (https://github.com/Hyx20/2026IMO). Supplementary materials (methods, figures, tables, graphical abstract, slides, videos, Chinese translated version, and updated materials) may be found in the online DOI or iMetaOmics Science http://www.imeta.science/imetaomics/.
