# Supplementary material for: The mediating roles of gut microbiota in the associations of air pollution and meteorological factors with metabolic syndrome
Source: IMetaOmics. 2026 May 7;3(2):e70099. doi: 10.1002/imo2.70099 (PMC13674454; doi:10.1002/imo2.70099)
Supplement: Supplementary file 1 [file IMO2-3-e70099-s002.docx]

Supporting Information to

**The mediating roles of gut microbiome in the associations of air pollution and meteorological factors with metabolic syndrome**

**Running title**: Gut microbiome, air pollution-meteorology, and metabolic syndrome

Yixiang Huang^1, 2, 3#^, Wei Wu^4#^, Yuan Zheng^1, 2, 3^, Hongwei Tu^4^, Yiping Duan^1,3^, Qijiong Zhu^1, 2, 3^, Zhiqing Chen^1, 2, 3^, Siwen Yu^1, 2, 3^, Yayi Li^1, 2, 3^, Wan Peng^1, 2, 3^, Wenjun Ma ^2*^, Tao Liu^1, 3*^

^1^Department of Public Health and Preventive Medicine, School of Medicine, Jinan University, Guangzhou 510632, China

^2^China Greater Bay Area Research Center of Environmental Health, School of Medicine, Jinan University, Guangzhou 510632, China

^3^Key Laboratory of Viral Pathogenesis & Infection Prevention and Control, Jinan University, Ministry of Education, Guangzhou 510632, China

^4^Guangdong Provincial Institute of Public Health, Guangdong Provincial Center for Disease Control and Prevention, Guangzhou 511430, China

^#^These authors contributed equally: Yixiang Huang, Wei Wu

*Correspondence: gztt_2002@163.com (Tao Liu), mawj@gdiph.org.cn (Wenjun Ma)

**METHODS**

**Study design and population**

This study included participants aged 18 years or older who participated in the Guangdong Gut Microbiome Project (GGMP) in Guangdong Province, China. Fourteen districts and counties were randomly selected from these regions using a probability proportional to size (PPS) sampling method. From each selected district or county, three streets or towns were randomly chosen, and from each street or town, two communities or villages were selected. Within each selected community or village, 45 households were randomly chosen to recruit family members aged 18 years and older. All family members over the age of 18 who consented to participate are surveyed. Participants is excluded from this survey if any of the following conditions apply: (i) they resided in their home for less than six months in the year before the survey; (ii) they were not living in the household during the survey period; (iii) they were pregnant; (iv) they were unable to participate due to serious illness, disability, or hospitalization; or (v) they were unwilling to participate for any other reason. In this study, 9172 individuals were initially investigated, with 6785 participants ultimately included after the exclusion of those who improperly provided stool or blood samples, failed to complete questionnaires, or submitted stool sample sequences yielding fewer than 10,000 reads, as well as those lacking necessary metabolic syndrome (MetS) diagnostic metadata. This study was approved by the Ethical Review Committee of the Chinese Center for Disease Control and Prevention (approval number: 201519-B). All participants provided written informed consent.

**Data collection**

The public health practitioners completed a face-to-face interview for each participant using a structured questionnaire, including demographic characteristics, lifestyle information, disease history, blood parameters, diet, medications, and home address. The anthropometric indicators, including waist circumference, weight, height, systolic blood pressure (SBP), and diastolic blood pressure (DBP), were measured using standard methods [1].

**Biological sample collection and testing**

Fasting blood and stool samples were collected for each participant according to standard protocols [1]. The collected fasting blood samples were used to measure the fasting blood glucose (FBG), triglycerides (TG), and high-density lipoprotein cholesterol (HDL-C). Each participant provided a stool sample and recorded their Bristol Stool Score at the time of sample collection. The gut microbiome was analyzed by sequencing the V4 region of the 16S rRNA gene.

**Outcome measurement**

MetS was defined as the presence of at least three of the following five criteria, according to the Chinese guidelines for the prevention and treatment of type 2 diabetes: (1) Waist circumference > 90 cm (males) or > 85 cm (females); (2) FBG ≥ 6.1 mmol/L (110 mg/dL) or a previous diagnosis of diabetes mellitus; (3) SBP/DBP ≥ 130/85 mmHg or a previous diagnosis of hypertension; (4) TG ≥ 1.7 mmol/L (150 mg/dL); (5) HDL-C < 1.04 mmol/L (40 mg/dL) [2].

**Assessment of air pollution and weather exposure**

We estimated individual-level weekly average exposure to ambient air pollutants (including PM_2.5_, NO_2_, sulfur dioxide (SO_2_), ozone (O_3_), carbon monoxide (CO)), and ambient temperature using a spatiotemporal land-use regression (LUR) model. Briefly, we constructed a spatiotemporal LUR model incorporating seven predictors: population density, road length, land use data (farmland, blue space, residential land, and green space), and environmental visibility. We then incorporated the residential addresses of each participant into the model to predict weekly average exposure levels of ambient air pollutants and temperature. Finally, we calculated the two-year average exposure levels of ambient air pollutants and temperature prior to the investigation date based on the weekly average concentrations (Table S5 and Figure S5) [3,4].

**Covariates**

Covariates were selected a priori based on their potential to confound the relationship between environmental exposures and MetS, as identified in the literature [5,6]. These covariates included sex (male or female), age (< 30, 30–39, 40–49, 50–59, 60–69, or ≥ 70 years), race (Han or Minority), annual household income (< 30, 30–50, 50–100, ≥ 100 × 1000 CNY or Refuse to answer or don’t know), years of education (0, 1–6, 7–9, 10–12, or ≥ 13 years), marital status (single, married, or others), occupation (manual worker, business or service staff, government official and clerk, professionals, unemployed, retiree, or others), type of home cooking fuel (clean or non-clean fuel), smoking status (nonsmoker, former smoker, current smoker (< 1 cigarette/day) or current smoker (≥ 1 cigarette/day)), alcohol consumption (no, < 3 days/week, 3–6 days/week, or every day), physical activity (< 20, 20–50, 50–100, 100–150 or ≥ 150 MET-h/week), urban–rural location (rural or urban), and season of survey (warm or cold) (Table S6). The average weekly intake levels of grains, vegetables, fruits, and meat products in the past 12 months were also included as covariates. Intake was assessed with a food-frequency questionnaire that recorded consumption frequency and portion size for each item.

**Statistical analysis**

The raw Illumina sequences were analyzed mainly using QIIME software (version 1.9.1) with the Greengenes database [1]. Categorical variables were compared between participants with and without MetS using Chi-square tests, while continuous variables were compared between the two groups using *t*-tests or Wilcoxon rank sum tests. Random forest models were applied to screen air pollutants associated with MetS, using the mean decrease in Gini to rank candidates. We ranked air pollutants by variable importance and selected PM_2.5_ and NO_2_ as the primary particulate and gaseous exposures (Figure S6). As a robustness check, we fitted an XGBoost model and used SHAP values to confirm the stability of the pollutant ranking and the direction of feature effects (Figure S7). In addition, ambient temperature was included as the primary meteorological exposure of interest, based on prior evidence linking temperature variability to metabolic health [7,8].

We used the Shannon index, the Chao1 index, the Observed species, and the PD whole tree to measure the alpha diversity of the gut microbiome. The Chao1 index and Observed species count primarily reflect microbial richness, the Shannon index captures both richness and evenness, and the PD whole tree reflects phylogenetic diversity. To characterize beta-diversity patterns, we performed principal coordinates analysis (PCoA) on Bray-Curtis dissimilarities computed from genus-level relative abundances. The top 50 principal coordinate axes were then used as input to Uniform Manifold Approximation and Projection (UMAP) to obtain a low-dimensional embedding that preserves local neighborhood structure and captures major patterns of community variation. K-means clustering was performed in the UMAP space using Euclidean distance, with the number of clusters (k = 4) determined by the elbow criterion based on the within-cluster sum of squares curve. These clusters were subsequently used as nodes to infer putative community transition trajectories with Slingshot, designating the cluster with the lowest MetS prevalence as the root and estimating a principal lineage along which each sample received a pseudotime value summarizing the major pattern of community compositional change.

To identify cluster-enriched genera, we compared genus-level abundances in each cluster with those in all other samples. A small pseudocount was added to the genus-level relative abundance data before centered log-ratio (CLR) transformation. Group differences were then assessed using two-sided Wilcoxon rank-sum tests. Effect sizes were summarized as differences in CLR-transformed abundances (ΔCLR). Multiple testing was controlled using the Benjamini-Hochberg method, which could reduce the likelihood of false positives. Genera with false discovery rate (FDR) adjusted *p* values < 0.05 and |ΔCLR| > 1 were considered enriched in a given cluster. Linear discriminant analysis (LDA) coupled with effect size analysis (LEfSe) was employed to identify significantly different abundant genera between the MetS group and the non-MetS group. Based on the genera highlighted by LEfSe, we further constructed an exploratory correlation-based co-variation network. Before network analysis, we added a small pseudocount to the genus-level relative abundances and performed CLR transformation. We then calculated correlations using the CLR-transformed data. Correlations with |ρ| ≥ 0.4 and FDR-adjusted *p* values < 0.05 were retained to construct the network. Additionally, we performed univariable permutational multivariate analysis of variance (PERMANOVA) using the Adonis function in the vegan package for R to descriptively assess the proportion of gut microbiome compositional variance associated with each host or environmental factor separately. The Adonis function was applied with 999 permutations and microbiome beta diversity based on Bray-Curtis dissimilarity for each factor.

We used generalized linear models to estimate associations between environmental exposure factors and gut microbial genera after adjusting for potential confounders. We further applied multivariable logistic regression models, with the same set of covariates, to evaluate associations between environmental exposures and MetS. Associations were identified based on nominal significance (*p* < 0.05) and study-wide significance, with a FDR threshold of less than 0.05 (FDR < 0.05). Mediation analysis was conducted under a counterfactual framework among genera identified in the preceding screening analyses to examine whether the associations between each environmental exposure and MetS could be statistically decomposed through these selected genus-level microbiome features. Analyses were implemented using the R package mediation. Before modelling, genus-level relative abundances were log-transformed to reduce skewness and improve model stability. For mediation analyses, *p* values were controlled using FDR, and the proportion mediated (Prop) was calculated on the logit scale as the ratio of the estimated indirect component to the total component. Prop was interpreted as an exploratory, scale-dependent decomposition of associations given odds-ratio non-collapsibility.

All statistical analyses were conducted using R software (version 4.3.0). All statistical tests were two-sided, and the *p-*value < 0.05 was considered significant.

**REFERENCE**

1. He, Yan, Wei Wu, Hui-Min Zheng, Pan Li, Daniel McDonald, Hua-Fang Sheng, Mu-Xuan Chen, et al. 2018. “Regional variation limits applications of healthy gut microbiome reference ranges and disease models.” *Nature Medicine* 24: 1532–1535. https://doi.org/10.1038/s41591-018-0164-x

2.Chinese Diabetes Society. 2021. “Guideline for the prevention and treatment of type 2 diabetes mellitus in China (2020 edition).” *Chinese Journal of Endocrine and Metabolism* 37: 311–398. https://doi.org/10.3760/cma.j.cn311282-20210304-00142

3. Liu, Xin, Yufeng Ye, Yi Chen, Xiaona Li, Baixiang Feng, Ganxiang Cao, Jianpeng Xiao, et al. 2019. “Effects of prenatal exposure to air particulate matter on the risk of preterm birth and roles of maternal and cord blood LINE-1 methylation: A birth cohort study in Guangzhou, China.” *Environment International* 133: 105177. https://doi.org/10.1016/j.envint.2019.105177

4. Liu, Tao, Xiaojiao Chen, Yanjun Xu, Wei Wu, Wenli Tang, Zihui Chen, Guiyuan Ji, et al. 2019. “Gut microbiota partially mediates the effects of fine particulate matter on type 2 diabetes: Evidence from a population-based epidemiological study.” *Environment International* 130: 104882. https://doi.org/10.1016/j.envint.2019.05.076

5. Liu, Dangzhen, Haiyan Zhao, Yiwen Zhang, Jingqing Hu, Hong Xu. 2025. “Efficacy and safety of berberine on the components of metabolic syndrome: a systematic review and meta-analysis of randomized placebo-controlled trials.” *Frontiers in Pharmacology* 16: 1572197. https://doi.org/10.3389/fphar.2025.1572197

6. Zhu, Zhanghang, Zongming Yang, Xinhan Zhang, Luhua Yu, Dandan Yang, Fanjia Guo, Lin Meng, et al. 2023. “Association of walkability and NO_2_ with metabolic syndrome: A cohort study in China.” *Environment International* 171: 107731. https://doi.org/10.1016/j.envint.2023.107731

7. Wang, Qiong, Changchang Li, Yanfang Guo, Adrian G. Barnett, Shilu Tong, Dung Phung, Cordia Chu, Keith Dear, Xuemei Wang, Cunrui Huang. 2017. “Environmental ambient temperature and blood pressure in adults: A systematic review and meta-analysis.” *Science of The Total Environment* 575: 276–286. https://doi.org/10.1016/j.scitotenv.2016.10.019

8. Fan, Ping, Xiaowei Xue, Jialu Hu, Qingxia Qiao, Tingting Yin, Xiaoling Yang, Xiyin Chen, Yuemei Hou, Renjie Chen. 2023. “Ambient temperature and ambulatory blood pressure: An hourly-level, longitudinal panel study.” *Science of The Total Environment* 864: 160854. https://doi.org/10.1016/j.scitotenv.2022.160854

**Figure S1 Radar plot showing the prevalence of MetS and its components in the overall population and stratified by sex.** Abbreviation: MetS, metabolic syndrome; BP, blood pressure; FBG, fasting blood glucose; TG, triglycerides; HDL-C, high-density lipoprotein cholesterol.

**Figure S2 Boxplots comparing long-term exposure levels to PM_2.5_, NO_2_, and ambient temperature between MetS and non-MetS participants.** Group differences were assessed using the Wilcoxon rank-sum test. ** p <* 0.05, *** p <* 0.01, **** p <* 0.001.

**Figure S3 Phylum and genus -level relative abundance of gut microbiota in non-MetS and MetS groups, based on 16S rRNA gene sequencing.**

**Figure S4 Elbow plot for selecting the optimal number of gut microbial community clusters.**

**Figure S5 The process of long-term exposure assessment to air pollutants.**

**Figure S6 Random forest variable-importance ranking of six ambient air pollutants (PM_2.5_, NO_2_, CO, PM_10_, SO_2_, and O_3_) for predicting MetS.**

**Figure S7 SHAP-based feature importance of six ambient air pollutants in the XGBoost model for predicting MetS.**
